# Supplementary material for: Vascular physiology drives functional brain networks
Source: Neuroimage. 2020 Aug 15;217:116907. doi: 10.1016/j.neuroimage.2020.116907 (PMC7339138; doi:10.1016/j.neuroimage.2020.116907)
Supplement: Multimedia component 1 [file mmc1.pdf]

Bright et al. – *Vascular physiology drives functional brain networks*

Supplemental Figures:

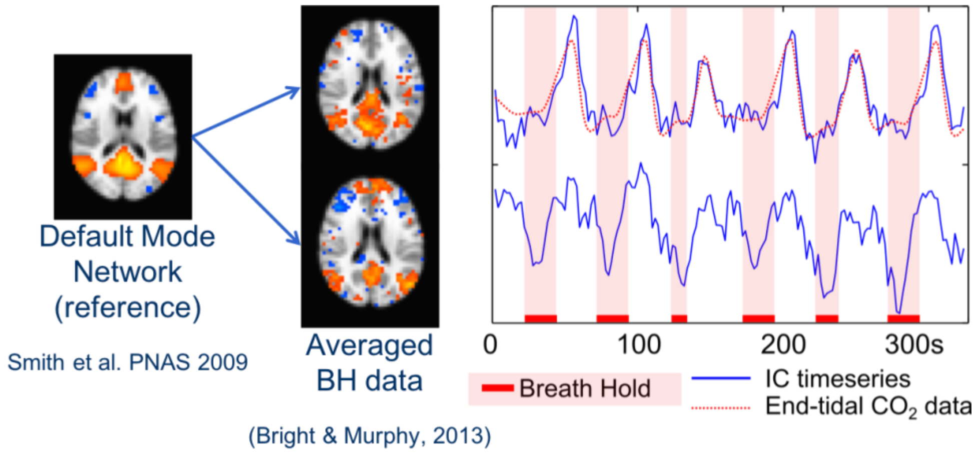

Figure S1. Decomposition of averaged breath-hold fMRI data using ICA resulted in the Default Mode Network being represented in two components. The component maps and associated time-series (blue) are presented, with the breath-hold timing and average end-tidal CO_2 trace (red). The top component shows BOLD signal increases closely following the end-tidal CO_2 trace. The bottom component shows BOLD signal decreases preceding and during the breath-hold stimulus, possibly reflecting deactivation of the neuronal Default Mode Network during the execution of the paced-breathing and breath-hold task. These results drove our hypothesis that functional brain networks, like the Default Mode Network, may be comprised of both neuronal and vascular systems.

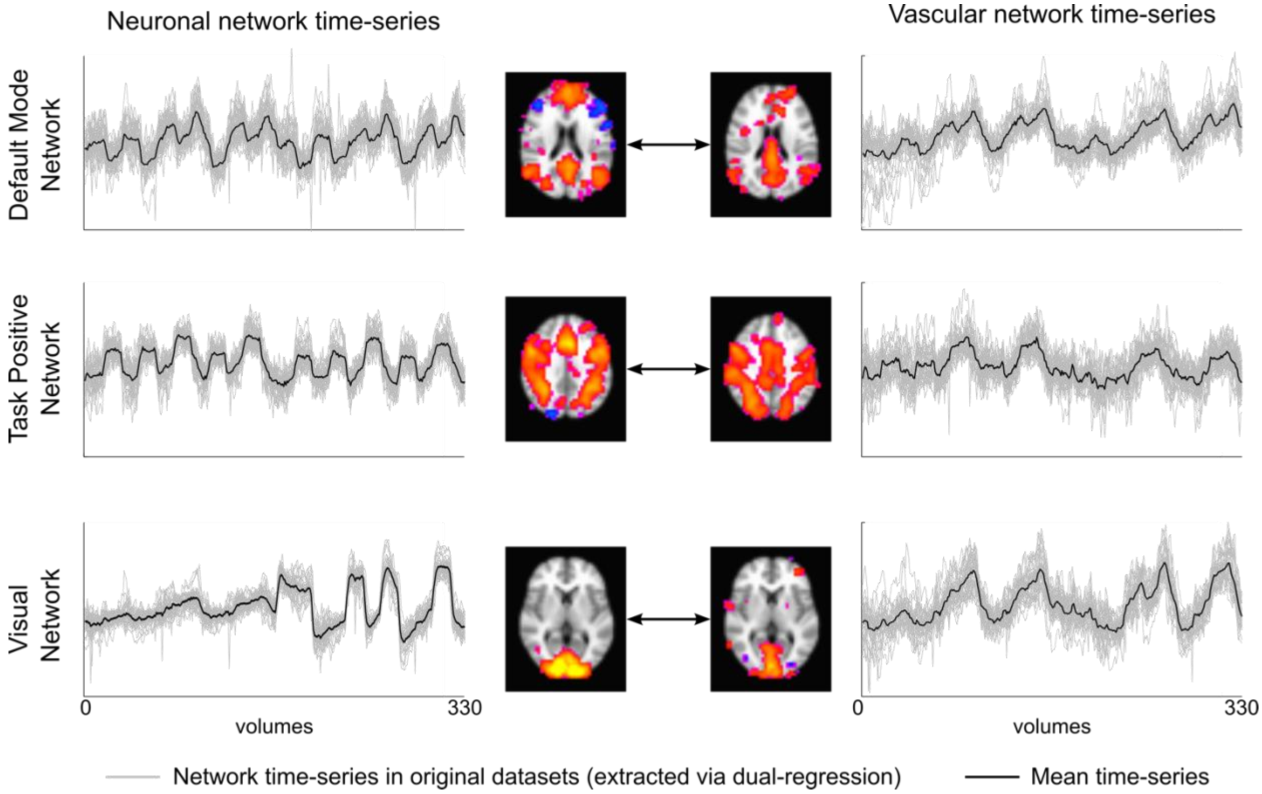

Figure S2. Time-series for vascular-neuronal network pairs. Time-series were extracted from the original 30 datasets using dual-regression. Networks of the left were identified via temporal correlation with the neural stimuli. Networks on the right were identified as having similar spatial characteristics to the neuronal network maps.

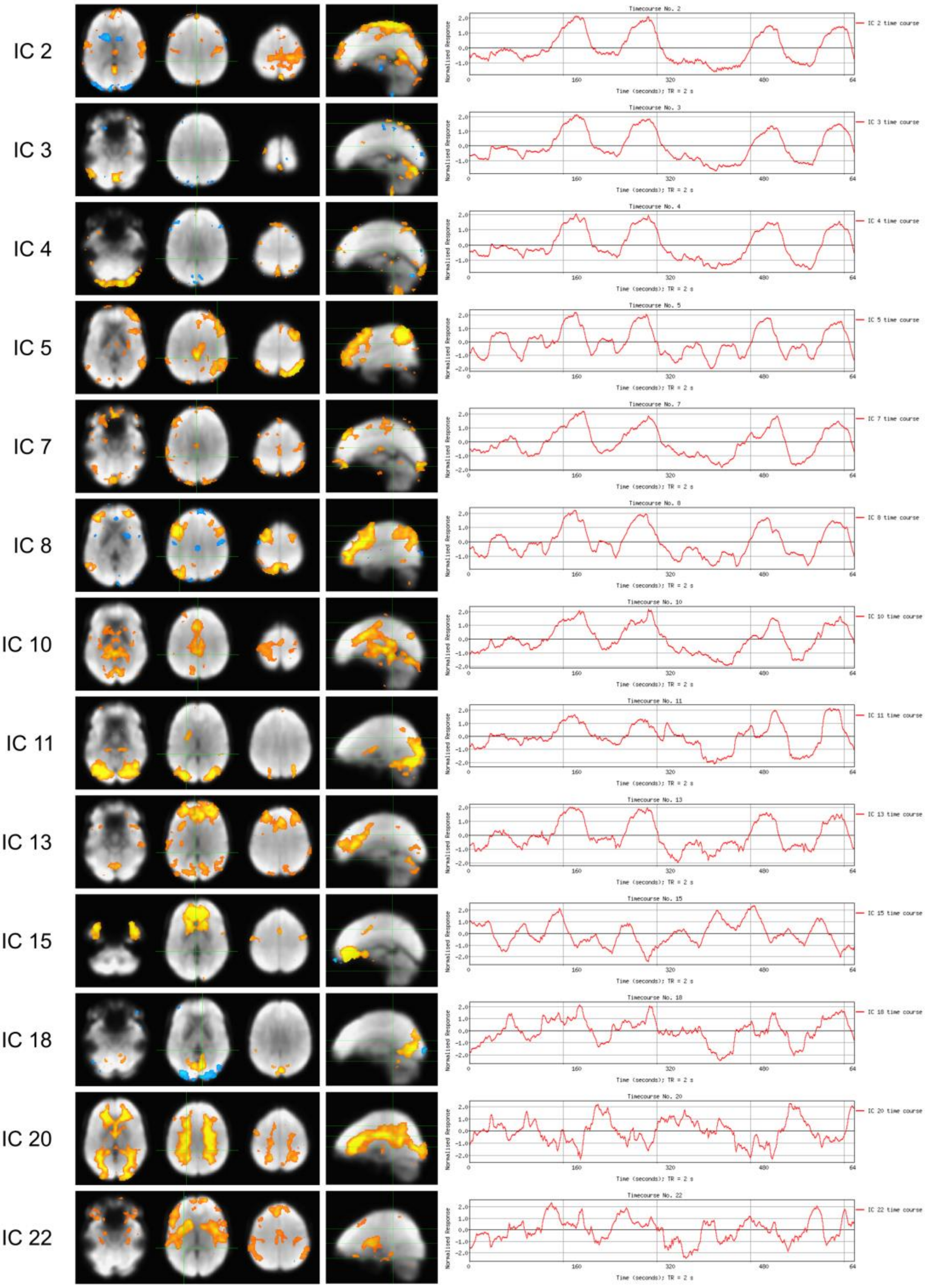

Figure S3. Additional components resulting from the Independent Component Analysis decomposition of the averaged dataset (fixed dimensionality of 30 components). Independent Components (ICs) that are discussed in the manuscript or without clear spatial structure are excluded from visualization. Several components reflect large vessel or CSF effects in both their spatial pattern and temporal signal properties (e.g., ICs 2, 3, 4, and 7). Numerous other ICs demonstrate spatial maps that could potentially reflect, at least in part, neural network structure (ICs 5, 8, 11, 13, 15); the temporal signals in these ICs reflect varying mixtures of vascular and neural stimulus designs. Of note, IC 18 (positive and negative responses in the occipital lobe), IC 20 (predominantly white matter regions) and IC 22 (predominantly subcortical gray matter regions) do not reflect any of the stimulus paradigms in the experimental protocol as clearly.

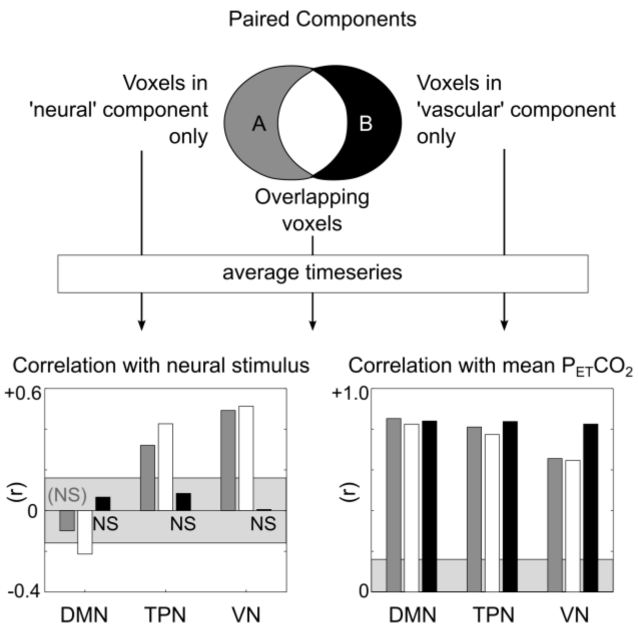

Figure S4. Characterization of common and unique voxels in the “more neural” and “more vascular” component pair. The average timeseries was extracted from the voxels unique to each network and common to both networks, and the correlation between each timeseries and the neural and CO_2 stimulus models was determined. The neural stimulus is strongly correlated with the voxels unique to the “neural” network and voxels common to both networks. The voxels unique to the “vascular” component are not significantly correlated with the neural stimulus design. In contrast, all timeseries are strongly correlated with the CO_2 model, reflecting the global nature of the vasodilatory stimulus.

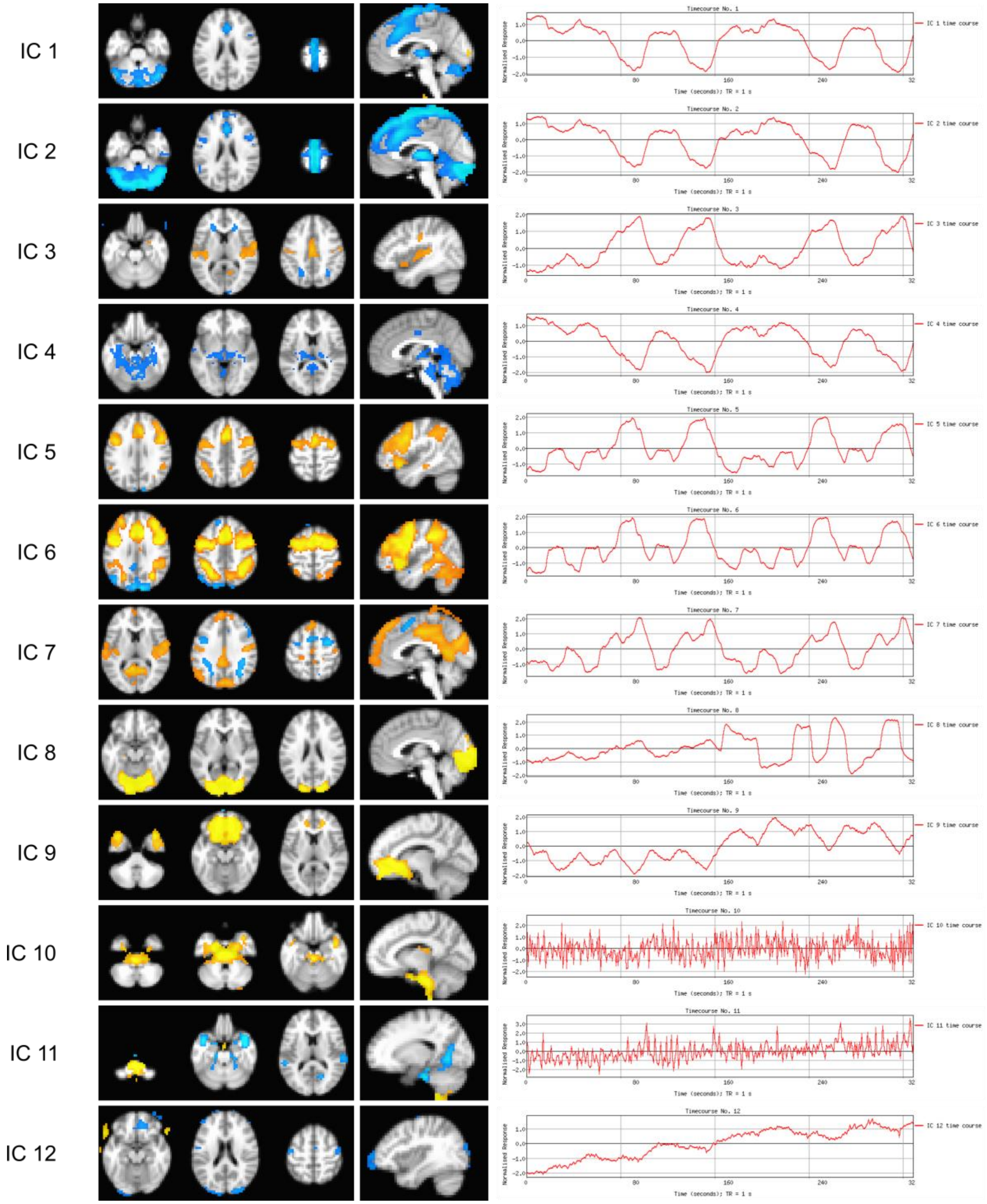

Figure S5. Results of Tensorial Independent Component Analysis decomposition (as implemented in MELODIC v3.15, part of FSL (FMRIB's Software Library, www.fmrib.ox.ac.uk/fsl) [Beckmann 2005]. Dimensionality was automatically estimated, resulting in 12 independent components visualized in the figure. Note that polarity of the main hypercapnia effect is often inverted (e.g., ICs 1, 2, and 4). Two components exhibit spatial structure similar to the expected task positive network (ICs 5 and 6), showing very similar timecourses reflecting both the working memory and gas paradigms. The visual network is isolated to one component (IC 8). Interestingly, no component cleanly reflects the spatial structure of the default mode network.

IC #	N-back Stimulus	Visual Stimulus	CO2 stimulus	Description
1	0.10	0.05	0.89	
2	-0.13	-0.07	-0.87	
3	-0.09	0.26	0.85	
4	-0.17	-0.02	-0.86	
5	-0.04	-0.08	0.89	
6	-0.35	-0.03	-0.84	TPN
7	0.03	0.24	0.85	
8	-0.31	0.04	0.83	
9	-0.03	0.56	0.73	VN
10	0.16	-0.01	0.92	
11	0.37	0.36	0.78	TPN
12	0.03	-0.16	0.89	
13	-0.38	-0.02	0.81	other
14	-0.25	0.13	-0.87	
15	0.41	-0.06	0.84	TPN partial
16	-0.18	0.81	0.43	VN
17	0.02	-0.16	-0.88	
18	-0.41	-0.02	0.77	TPN
19	0.57	-0.07	0.78	TPN
20	-0.11	-0.37	-0.77	
21	0.82	-0.14	0.54	TPN
22	-0.08	0.13	0.90	
23	0.34	-0.45	0.58	TPN partial
24	-0.12	-0.43	-0.66	other
25	-0.33	0.04	-0.54	other
26	-0.23	0.16	0.08	
27	-0.18	0.28	-0.25	
28	-0.04	0.30	-0.02	
29	0.00	-0.11	-0.22	
30	-0.12	-0.45	-0.32	other

shaded cells: **|r|>0.3** **|r|>0.3** **|r|<0.7**

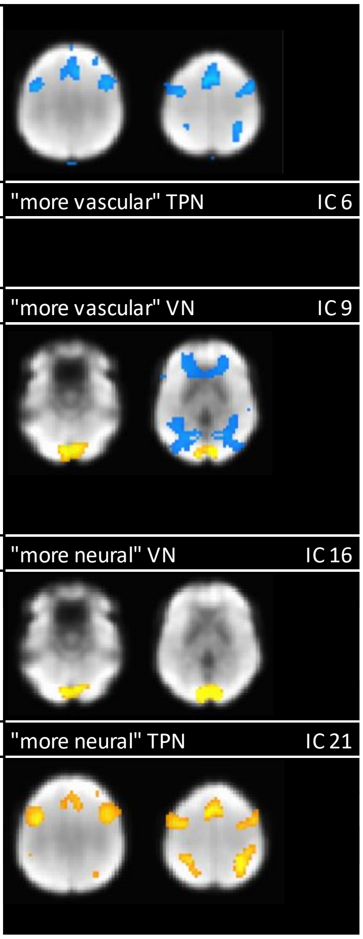

Figure S6. Results of Tensor ICA decomposition of the preprocessed 30 datasets. The correlation coefficient between the component timeseries and the neural and CO_2 stimulus timeseries are provided. Blue shaded values highlight robust correlations (|r|>0.3) with the n-back and visual task. Gray shaded values indicate components with a less robust (|r|<0.7) correlation with the CO_2 task. Pairs of "more neural" and "more vascular" TPN and VN networks are identified. Several additional components reflect spatial patterns similar to the TPN or certain nodes of the TPN. No component demonstrated spatial patterns corresponding to the DMN. Note, Tensor ICA did not preserve polarity of signal changes consistently. Isolating negative correlations with the n-back task cannot be performed as in the single ICA decomposition of the averaged dataset.

A)

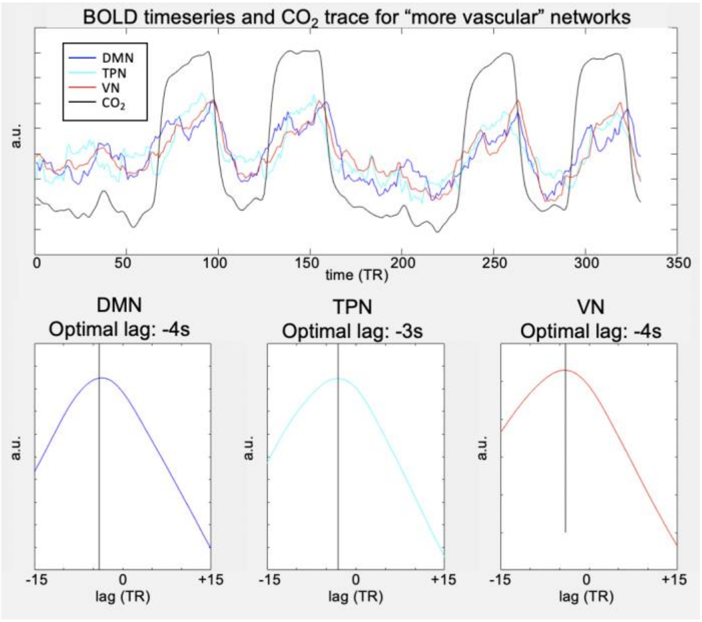

B)

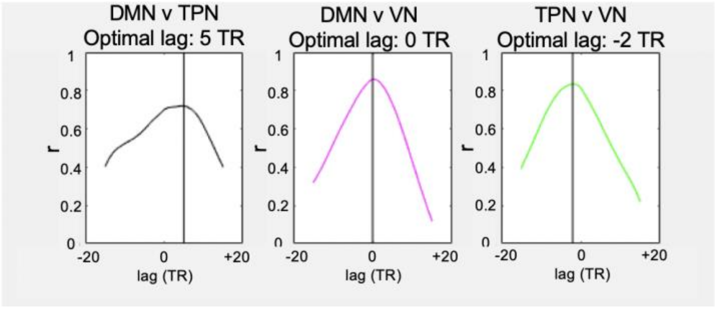

Figure S7. Analysis of temporal lag effects contributing to distinct timeseries of "vascular" components. **A)** Timeseries of the three "more vascular" components reflecting the DMN, TPN, and VN network structure, and the results of cross-correlation between these time-series and the CO_2 timeseries (averaged across datasets). The optimal lag (corresponding to peak correlation) is indicated for each network. **B)** Cross-correlation between each "vascular" network timeseries with optimal lag indicated.

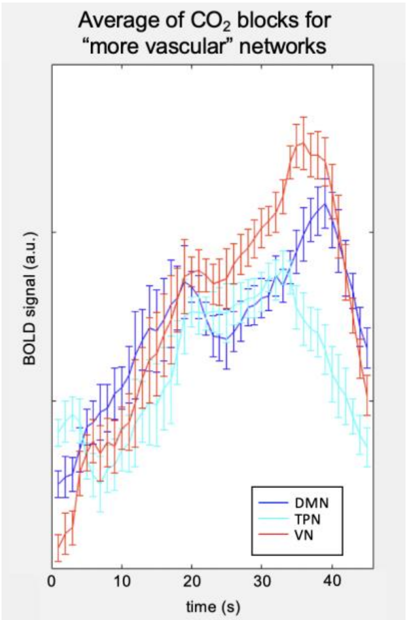

Figure S8. Block average over the hypercapnic periods for the three "more vascular" component timeseries. Clear differences in the response shape/dynamics are visible.
